# Supplementary material for: Circulating exosomes suppress the induction of regulatory T cells via let-7i in multiple sclerosis
Source: Nat Commun. 2018 Jan 2;9:17. doi: 10.1038/s41467-017-02406-2 (PMC5750223; doi:10.1038/s41467-017-02406-2)
Supplement: Supplementary file 1 — Supplementary Information [file 41467_2017_2406_MOESM1_ESM.pdf]

Supplementary Figure 1

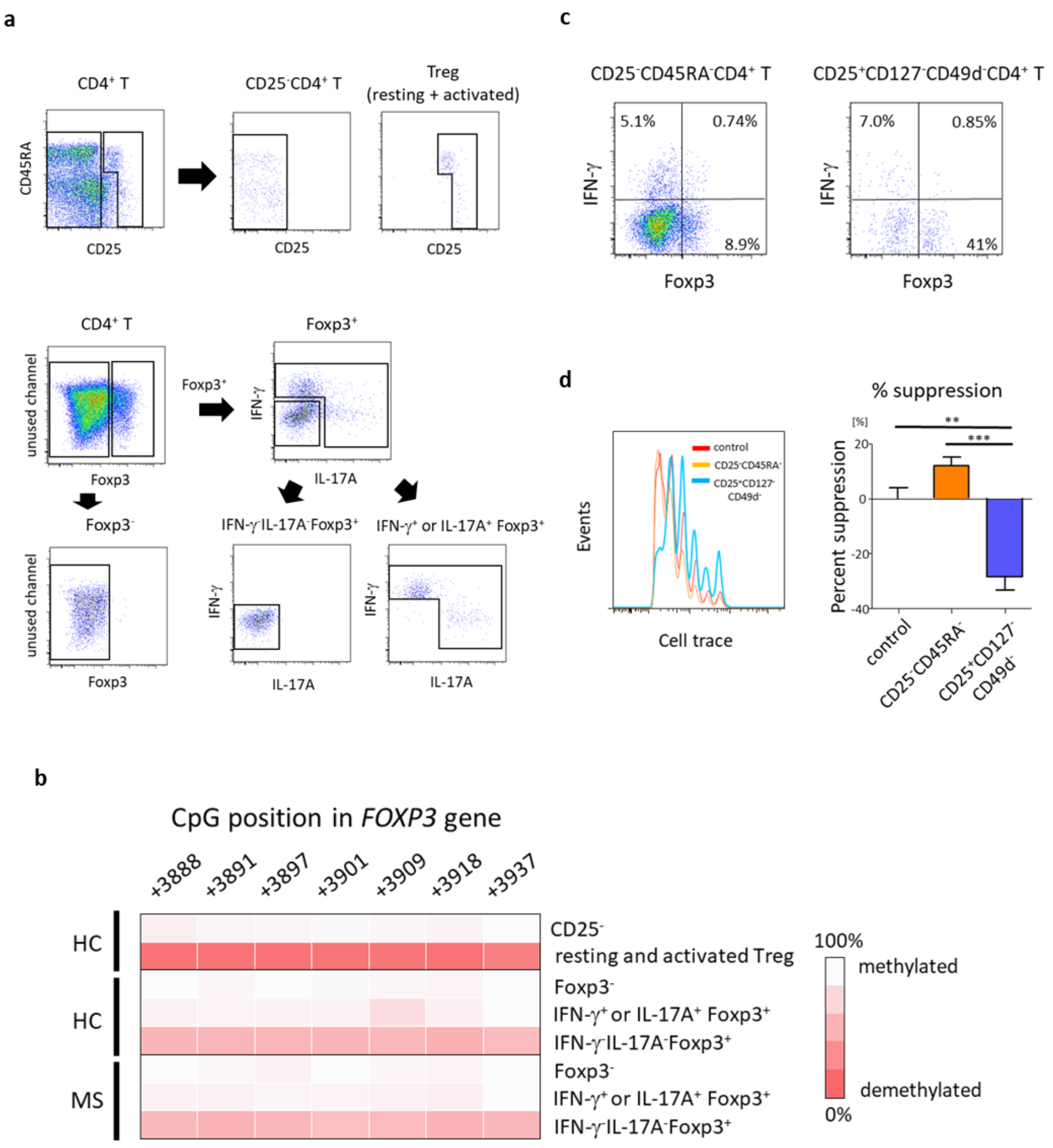

Supplementary Figure 1. Potent regulatory T cells are discriminated based on the secretion of IFN-γ or IL-17A among Foxp3^{+}CD4^{+} T cells.

(a) *Upper*: Gating strategy for isolation of CD25^{-}CD4^{+} T cells, resting Treg cells (CD45RA^{+}CD25^{+}), and activated Treg cells (CD45RA^{-}CD25^{high}) from primary non-stimulated

$CD4^+$ T cells. *Lower*: Gating strategy for isolation of $Foxp3^-CD4^+$ T cells, IFN-γ^-IL-$17A^-$ $Foxp3^+CD4^+$ T cells, and $Foxp3^+CD4^+$ T cells secreting IFN-γ or IL-17A from $CD3^+$ T cells after stimulation with anti-CD3 and anti-CD28 mAbs for 72 h. (b) Analysis of DNA methylation status in the STAT5-responsive region of the *FOXP3* gene in each population sorted as in (a). $CD25^-CD4^+$ T cells, and resting and activated Treg cells were analysed as a negative and positive control according to a previous paper [1]. $CD25^-CD4^+$ T cells, and resting and activated Treg cells were obtained from one male HC. $Foxp3^-CD4^+$ T cells, IFN-γ^-IL-$17A^-Foxp3^+CD4^+$ T cells, and $Foxp3^+CD4^+$ T cells secreting IFN-γ or IL-17A were obtained from one male HC and one male patient with MS. Percentages of methylation of indicated CpG sites were described. (c) $CD25^-CD45RA^-CD4^+$ T cells and $CD25^+CD127^-CD49d^-CD4^+$ T cells were evaluated for the expression of Foxp3 and IFN-γ after stimulation with anti-CD3 and anti-CD28 mAbs for 48 h. IL-$17A^+$ population, which accounted for about 1 % of each subset, was excluded before this gating. (d) Suppression assay was performed using the population described in (c). $CD45RA^+CD25^-CD4^+$ T cells and $CD3^-CD56^-$ cells were used as responder cells and antigen presenting cells (APC), respectively. Responder cells were stained with a cell trace marker, and cultured with $CD25^-CD45RA^-CD4^+$ T cells or $CD25^+CD127^-CD49d^-CD4^+$ T cells or without any additional cells (control), in the presence of APCs under stimulation with anti-CD3 mAb for 120 h. The percentage of suppression was determined based on division indices. n = 3 or 4 in each group in (d). A one-way ANOVA with Bonferroni's comparison test was used for statistical analysis. Error bars represent the mean ± s.e.m. ** $p < 0.01$, *** $p < 0.001$. s.e.m.: standard error of the mean; ANOVA: analysis of variance; STAT5: signal transducer and activator of transcription 5.

Supplementary Figure 2

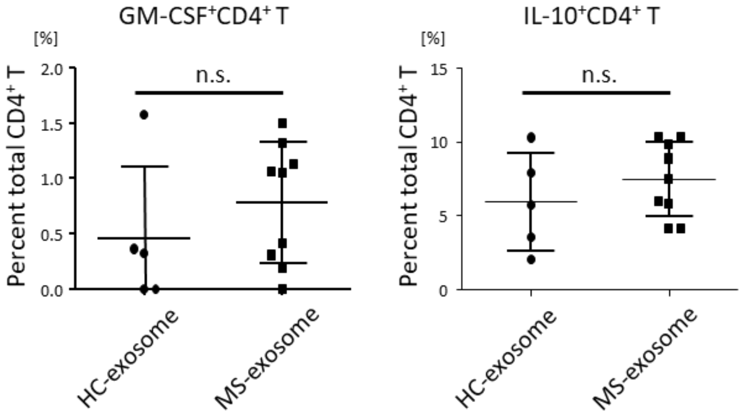

Supplementary Figure 2. GM-CSF$^+$ or IL-10$^+$ CD4$^+$ T cells are not affected after co-culture with MS patient-derived exosomes.

CD3$^+$ T cells were cultured with exosomes derived from HC (HC-exosome) or patients with MS (MS-exosome) under stimulation with anti-CD3 and anti-CD28 mAbs for 48 h. Subsequently, expression of intracellular cytokines in the T cells was analysed by flow cytometer. T cells cultured with HC-exosome and those with MS-exosome did not differ significantly in the frequency of GM-CSF$^+$ or IL-10$^+$ cells among CD4$^+$ T cells. An unpaired *t*-test was used for statistical analysis. Error bars represent the mean ± s.d. s.d.: standard deviation; n.s.: not significant; mAb: monoclonal antibody.

Supplementary
Figure 3

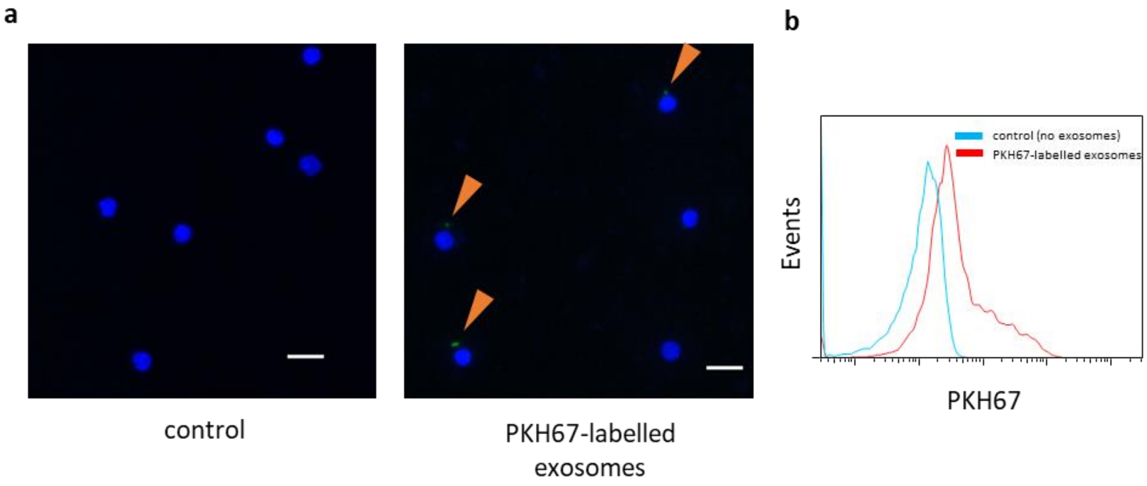

Supplementary Figure 3. Exosomes interact with T cells.

(a) Exosomes were isolated from HC and labelled with PKH67 green fluorescence lipid dye. A control fluorescence solution was prepared in the absence of exosomes, by the same procedure. T cells were co-cultured with labelled exosomes or control solution for 24 h, and then analysed by fluorescence microscopy. Arrowheads point to PKH67 green fluorescence on T cells with nuclei stained with DAPI (blue). Scale bar = 20 µm. (b) The fluorescence intensity of PKH67 in co-cultured T cells was analysed by flow cytometry.

Supplementary
Figure 4

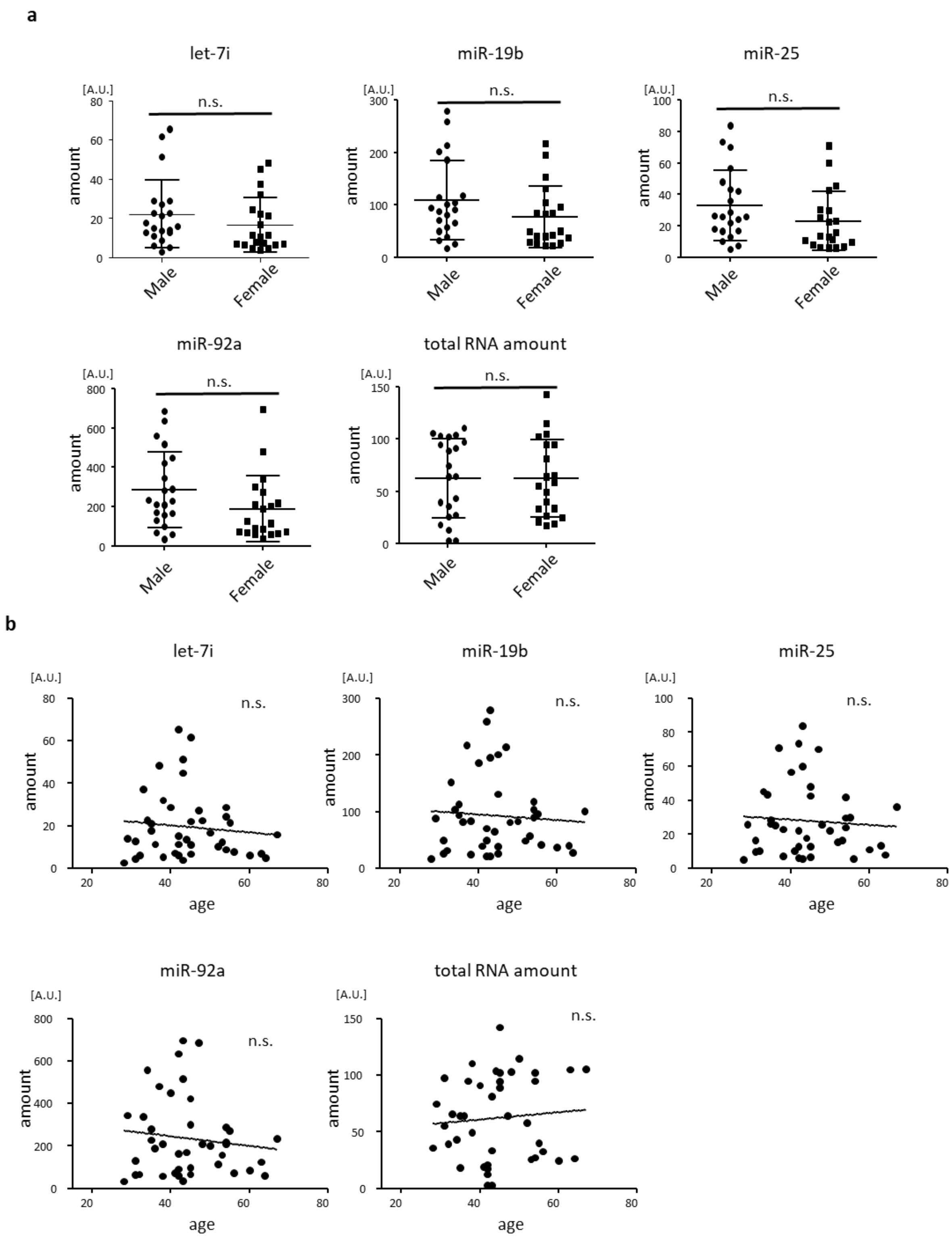

Supplementary Figure 4. MiRNA expression in the exosomes of patients with MS is not influenced by sex or age.

(a) Comparison between male and female. No significant differences were noted between male and female in the expression level of each miRNA as well as the amount of total RNA. (b) Correlation plot of age and miRNA expression. An unpaired *t*-test was used in (a), and Pearson's correlation analysis was used in (b) for statistical analysis. Error bars represent the mean ± s.d. s.d.: standard deviation; n.s.: not significant; A.U.: arbitrary unit.

Supplementary
Figure 5

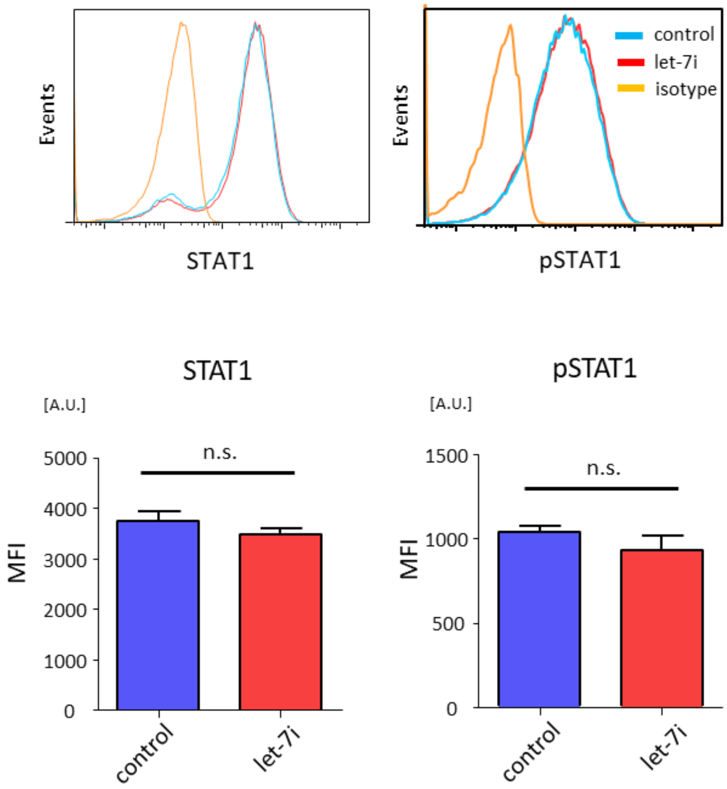

Supplementary Figure 5. Exogenously added *let-7i* shows no significant effect on the expression levels of STAT1 and pSTAT1 in $CD4^+$ T cells.

T cells were transfected with *let-7i* and then cultured under stimulation with anti-CD3 and anti-CD28 mAbs for 72 h. The expression levels of STAT1 and pSTAT1 in $CD4^+$ T cells were evaluated based on the MFI. The expression levels are shown by histograms at the top. n = 6 in each group. An unpaired *t*-test was used for statistical analysis. Error bars represent the mean ± s.e.m. s.e.m.: standard error of the mean; n.s.: not significant; A.U.: arbitrary unit; MFI: mean fluorescence intensity; STAT1: signal transducer and activator of transcription 1; pSTAT1: phosphorylated STAT1; mAb: monoclonal antibody.

Supplementary
Figure 6

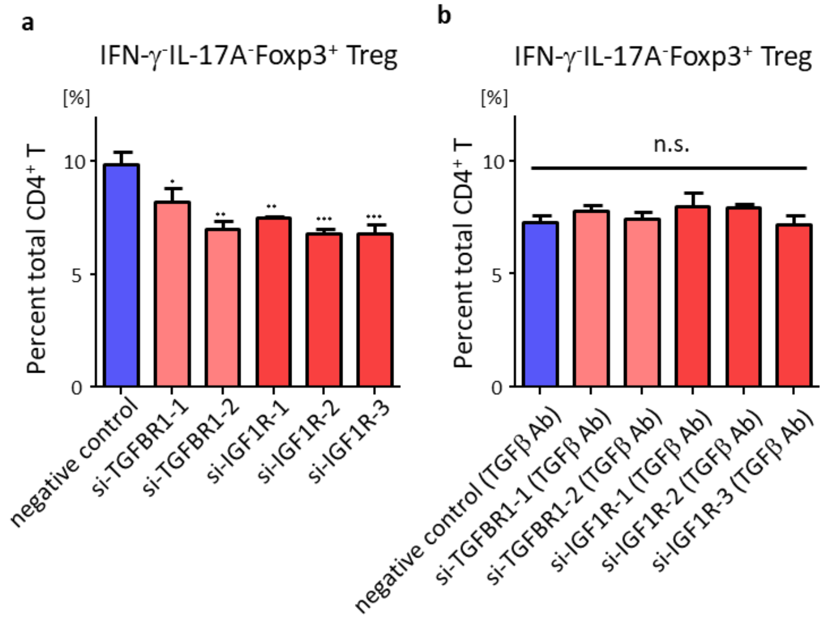

Supplementary Figure 6. Differences in IFN-γ^-IL-17A$^-$Foxp3$^+$CD4$^+$ Treg cell frequencies disappear by TGFβ neutralisation.

T cells were transfected with five different siRNAs targeting *TGFBR1* or *IGF1R*, or a negative control siRNA, and then cultured under stimulation with anti-CD3 and anti-CD28 mAbs for 72 h. Anti-TGFβ neutralising antibody or control mouse IgG (10 μg/ml) was added in the culture ((b) and (a), respectively). The Treg frequencies were compared between each siRNA group and the negative control siRNA group. Data are representative of two independent experiments. n = 3 in each group. A one-way ANOVA with Dunnett's comparison test was used for statistical analysis. Error bars represent the mean ± s.e.m. * $p < 0.05$, ** $p < 0.01$, *** $p < 0.001$. s.e.m.: standard error of the mean; n.s.: not significant; ANOVA: analysis of variance; IGF1R: insulin like growth factor 1 receptor; TGFBR1: transforming growth factor beta receptor 1.

Supplementary
Figure 7

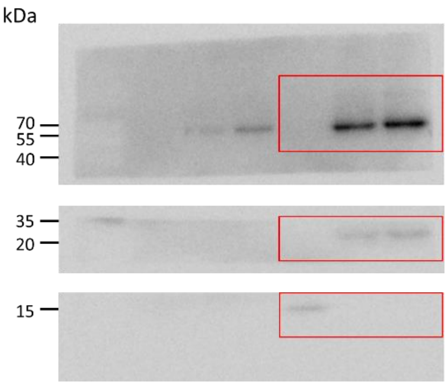

Supplementary Figure 7. The original blot image of Fig. 1b.

Red boxes indicate blot images that were used in Fig. 1b. To detect Cytochrome c, CD9, and CD63 simultaneously, blotting membranes were divided into three parts and incubated with an antibody against each protein (top: CD63; middle: CD9; bottom: Cytochrome c).

Supplementary Figure 8

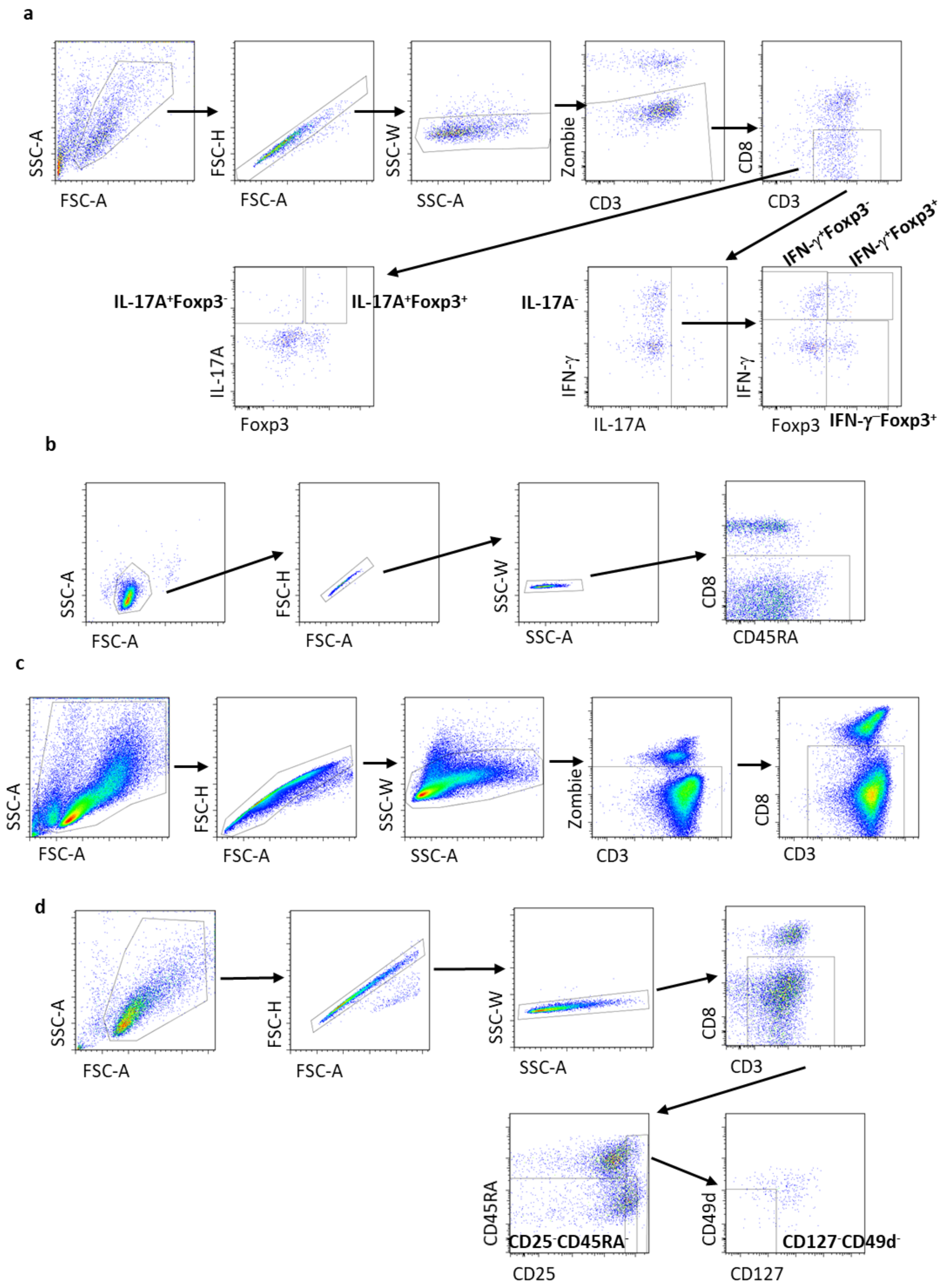

Supplementary Figure 8. Gating strategies used in this study.

(a) Gating strategy to examine inflammatory and regulatory $CD4^+$ T cell populations based on the expression of Foxp3, IFN-γ, and IL-17A. This strategy was used in Fig. 1d, and similar strategies were used in Fig. 4a, b, 5b, c, 6a, c, 7a, b, 8a, and Supplementary Fig. 6. (b) Gating strategy to identify $CD4^+$ T cells. This strategy was used in Fig. 5a. $CD3^+$ T cells were sorted in advance with an autoMACS Separator. A similar strategy was used in Supplementary Fig. 1a. (c) Gating strategy to identify $CD4^+$ T cells. This strategy was used in Fig. 6b. A similar strategy was used in Supplementary Fig. 5. (d) Gating strategy for isolation of $CD25^-CD45RA^-CD4^+$ T cells and $CD25^+CD127^-CD49d^-CD4^+$ T cells. This strategy was used in Supplementary Fig. 1c.

Supplementary Figure 9

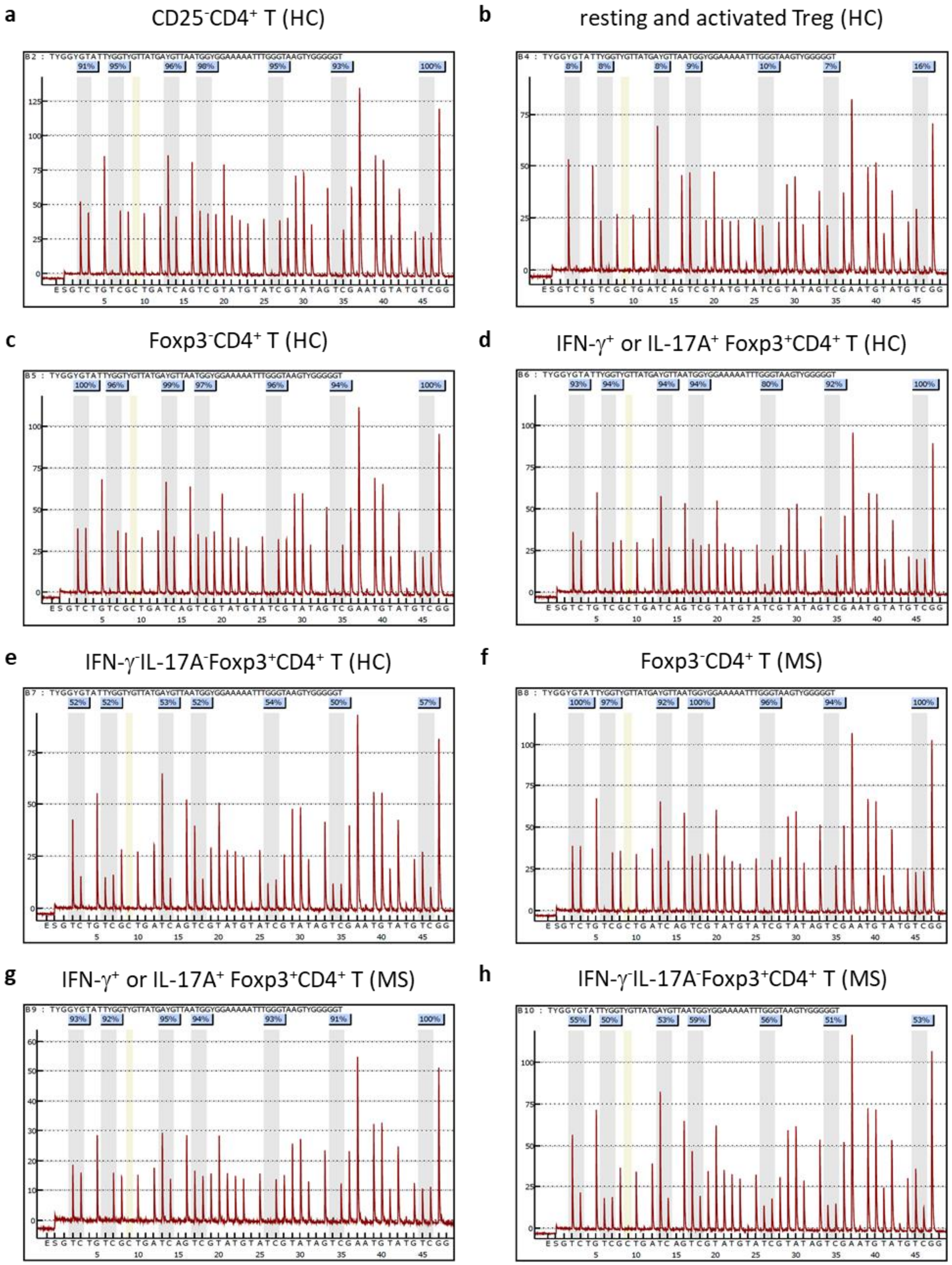

Supplementary Figure 9. Pyrosequencing data used in Supplementary Fig. 1b.

DNA methylation status in the STAT5-responsive region of the *FOXP3* gene was examined as described in Supplementary Fig. 1b. The obtained raw data are indicated. Resting and activated Treg cells were defined as $CD45RA^{+}CD25^{+}$ and $CD45RA^{-}CD25^{high}$ $CD4^{+}$ T cells, respectively. STAT5: signal transducer and activator of transcription 5.

Supplementary Table 1

	Microarray experiment		Validation cohort (miRNA)		Flow cytometry experiment (PBMC)	
Group	HC	MS	HC	MS	HC	MS
Number	4	4	14	27	13	26
Age (mean [SD])	36.5 [9.9]	33.3 [9.2]	41.1 [8.0]	46.0[10.5]	43.8 [10.1]	45.8 [10.6]
Sex (M / F)	2/2	2/2	9/5	12/15	6/7	10/16
RRMS / SPMS		4/0		21/6		19/7

Supplementary Table 1. Demographics of HC and patients with MS.

All the parameters were not significantly different between HC and patients with MS. PBMC: peripheral blood mononuclear cell; RRMS: relapsing-remitting MS; SPMS: secondary-progressive MS.

Supplementary Reference

1. Miyara, M., et al. Functional delineation and differentiation dynamics of human CD4+ T cells expressing the FoxP3 transcription factor. Immunity 30, 899-911 (2009).
